# RPS27A as a potential clock-related diagnostic biomarker for myocardial infarction: Comprehensive bioinformatics analysis and experimental validation

**DOI:** 10.1016/j.clinsp.2025.100677

**Published:** 2025-05-22

**Authors:** Rui Xu, Changshun Yan, GuiQiu Cao

**Affiliations:** Department of Cardiology, Fifth Affiliated Hospital of Xinjiang Medical University, China

**Keywords:** Bioinformatics, Circadian system, Clock genes, Myocardial infarction, RPS27A

## Abstract

**Background:**

The circadian system plays a crucial role in managing cardiovascular functions, with disturbances in this system associated with Myocardial Infarction (MI). Despite this connection, the exact mechanisms by which clock genes influence MI occurrence are not well-defined. This research focused on investigating the link between clock genes and MI.

**Methods:**

The authors examined MI microarray datasets (GSE151412 and GSE60993) from the GEO database, concentrating on Differentially Expressed Genes (DEGs) associated with the circadian system. To clarify critical biological functions and pathways, the authors performed enrichment analyses using Gene Ontology (GO) and the Kyoto Encyclopedia of Genes and Genomes (KEGG). Through Lasso regression, the authors pinpointed hub genes and confirmed their relevance using both the GSE66360 dataset and quantitative Reverse Transcription-Polymerase Chain Reaction (qRT-PCR). Furthermore, the authors conducted single-Gene Set Enrichment Analysis (GSEA) to reveal pathways linked to the hub gene. The analysis extended to exploring drug interactions and networks involving competing endogenous RNA (ceRNA).

**Results:**

The present analysis identified ten clock genes associated with circadian rhythms that showed differential expression between MI patients and healthy controls. Enrichment analysis suggested these genes’ roles in pathways like the Gap junction and circadian rhythm pathways. Following Lasso regression and validation, RPS27A was identified as the main hub gene. GSEA further highlighted enriched pathways, such as mismatch repair. Additionally, immune infiltration analysis revealed notable differences in B-cell and CD4+ T-cell populations between the MI group and the control group.

**Conclusion:**

The present findings suggest that the clock-related gene RPS27A is associated with MI, potentially influencing its development through circadian rhythm regulation. These results enhance the understanding of MI pathogenesis and may offer new avenues for therapeutic intervention.

## Highlights

• Clock genes are linked to myocardial infarction, offering novel insights into its pathogenesis.
• Ten differentially expressed clock genes suggest new pathways in myocardial infarction development.
• RPS27A’s core role in myocardial infarction was validated via Lasso regression and qRT-PCR, showing elevated expression.
• Gene enrichment analysis uncovers gap junction and circadian rhythm pathways in myocardial infarction.
• RPS27A was identified as a potential molecular target for diagnosing myocardial infarction.

## Introduction

Living organisms have evolved efficient mechanisms to interact with their environment. One crucial aspect of this interaction is the diurnal cycle, which influences various physiological processes regulated by internal circadian rhythms. These biological “clocks” enable organisms to anticipate and adapt to daily environmental shifts, preparing them for behaviors like periods of wakefulness and rest. Light serves as the primary Zeitgeber for circadian rhythms, detected by the retina and conveyed to the Suprachiasmatic Nucleus (SCN) within the hypothalamus, which functions as the master clock.^1^ In response, the SCN releases neural and hormonal cues, including melatonin, to align peripheral clocks found throughout most bodily tissues. These clocks operate through precisely regulated transcriptional and translational feedback loops that cycle approximately every 24 hours. The synchronization of these clocks allows organisms to optimize energy utilization by prioritizing essential processes during specific time periods and inhibiting others that are necessary at the opposite end of the circadian rhythm.^2^ However, these cyclical variations in physiology can affect the body's ability to manage stress.

Circadian rhythms are essential in controlling several cardiovascular functions, such as regulating blood pressure, heart rate, endothelial health, and clot formation. Disruptions in circadian rhythm can significantly impact the body, increasing the risk of developing heart disease. The intrinsic circadian rhythm of cardiac muscle cells may contribute to the time-dependent characteristics of cardiovascular physiology.^3^ Human myocardial infarctions demonstrate diurnal patterns, occurring more frequently during the early morning hours compared to evenings. The circadian clock significantly impacts the regulation of perioperative myocardial injury in patients undergoing aortic valve replacement.^4^ Recent research indicates that the timing of the day affects both the size of myocardial infarcts and left ventricular function after an ischemic event.^5^ Adverse cardiovascular events, such as acute heart failure, stroke, cardiac arrhythmias, and myocardial infarction, exhibit daily fluctuations, with increased prevalence in the early morning, although the specific underlying mechanism remains unclear. Understanding the intrinsic correlation between circadian rhythms and the occurrence of myocardial infarctions can provide novel insights for preventing and managing these events.

The circadian system is an internal biological rhythm that governs the physiology and behavior of organisms, evolving to synchronize with the 24-hour solar day cycle. This internal clock is regulated through fluctuating hormone and protein levels via negative feedback mechanisms. Core regulation relies on the transcription-translation feedback loop, which governs the expression of essential clock genes, including Period, Clock, Cryptochrome, and Bmal1. These genes not only generate distinct physiological and behavioral rhythms but also influence their own expression.^1^ In mice, mutations in clock genes have been found to impact circadian parameters, whereas genome-wide association studies in humans have revealed genetic variations linked to circadian systems.^2^^,^^6^ Individuals with cardiovascular diseases often exhibit disruptions in their biological rhythms. Evidence suggests that genetic variations in certain circadian clock genes may be linked to circadian phenotypes observed in cardiovascular disease patients.^3^

This study employed transcriptome sequencing and bioinformatics analysis to examine clock-related genes in individuals experiencing MI. Blood samples from MI patients were collected, and clock gene expression levels were confirmed using qRT-PCR. The primary goal was to explore new strategies for MI diagnosis and treatment.

## Materials & methods

### Data sources

This study analyzed three datasets from the GEO database. The first dataset, GSE141512, comprised whole blood samples from six MI patients and six healthy controls, with whole transcriptome sequencing performed on the GPL17586 platform.^7^ The second dataset, GSE60993, contained 7 healthy control samples and 17 MI samples, sequenced using the GPL6884 platform.^8^ The third dataset, GSE66360, was used for validation and comprised 49 healthy controls and 50 MI patient samples, with sequencing performed on the GPL570 platform.^9^

### Identification of clock-associated DEGs

In this study, the authors used the R package limma for data analysis, obtaining an expression matrix for circadian rhythm genes from the GSEA website (https://www.gsea-msigdb.org/gsea/downloads.jsp) that included 201 clock-related genes. Expression data from the GSE141512 and GSE60993 datasets were combined, followed by batch normalization with the SVA package in R to minimize differences between the datasets. The authors then performed a differential expression analysis to compare MI patients and controls, focusing on clock genes specifically. Genes with a p-value below 0.05 and a Fold Change (FC) greater than 1.0 were considered differentially expressed. A Venn diagram was generated to display the overlap between these DEGs and clock-related genes.

### Analysis of functional enrichment

To conduct the enrichment analyses for GO terms and KEGG pathways, the authors used the clusterProfiler R package. The authors established statistical significance criteria with a False Discovery Rate (FDR) of less than 0.05 and an adjusted p-value below 0.05. Gene Ontology (GO) terms were categorized into three main groups: Biological Process (BP), Molecular Function (MF), and Cellular Component (CC).

### Hub gene identification

Hub clock-associated DEGs were identified by comparing samples from control and MI patients. These DEGs were subsequently utilized for feature selection. To decrease data dimensionality, the authors applied the LASSO algorithm from the glmnet package, facilitating the identification of essential MI biomarkers. The hub clock-associated DEGs were subsequently validated in the independent dataset GSE66360, where the authors confirmed the identified hub genes.

### Recruitment of patients and sample collection

To confirm the biomarkers, qRT-PCR experiments were conducted using peripheral blood samples from patients diagnosed with MI. Participants were included if they met the following criteria: 1) 18 years of age or older, 2) Diagnosed with MI according to the European Society of Cardiology (ESC) guidelines, and 3) Onset of symptoms within 12 hours prior to undergoing Percutaneous Coronary Intervention (PCI). Exclusion criteria were: 1) Presence of cardiogenic shock, 2) Previous history of myocardial infarction or coronary artery bypass grafting, and 3) Severe liver disease. Samples were acquired from subjects who were registered in the Department of Cardiology of the Fifth Affiliated Hospital of Xinjiang Medical University. This research was authorized by the Medical Ethics Committee of the Fifth Affiliated Hospital of Xinjiang Medical University (n° XYDWFYLSH-2022028). Participants were first informed about the research and then provided written consent. In addition, this study is a clinical observational study, following the strengthening of the Reporting of Observational Studies in Epidemiology (STROBE) guidelines.

### qRT-PCR assay for validation of the hub genes

To verify the role of the biomarkers in MI, their expression levels were validated by Reverse Transcription quantitative Polymerase Chain Reaction (qRT-PCR). Total RNA was extracted from blood samples following the standard protocols provided by the RNA Extraction Reagent (Invitrogen, USA). The synthesis of complementary DNA (cDNA) was carried out using the 5X All-In-One RT MasterMix (abm, China). Polymerase Chain Reaction (PCR) amplification was performed on a CFX96 real-time PCR instrument (BIO-RAD, USA) with EvaGreen Express 2×qPCR MasterMix-Low Rox (abm, China). Reaction conditions were as follows: pre-denaturation: 95 °C for 3 min; amplification: 95 °C for 15 s, 60 °C for 60 s, 40 cycles. The PCR results were normalized to β-actin expression levels, and quantification was achieved using the 2^−ΔΔCt^ method. Detailed information about the primers used in these assays can be found in Table S1.

### Single-gene GSEA assessments for identified Hub genes

The single-gene GSEA method was employed to explore the relationships between pathways and the functions of hub clock-associated DEGs. Statistical significance was determined using the following criteria: a p-value of less than 0.05, Normalized Enrichment Scores (NES) exceeding 1, and False Discovery Rate (FDR) q-values below 0.25.

### Analysis of immune cell populations

ImmuCellAI (https://guolab.wchscu.cn/ImmuCellAI) is a tool designed to estimate the infiltration levels of 24 immune cell types using RNA-Seq data or gene expression profiles derived from microarray data. The normalized datasets (GSE141512 and GSE60993) were uploaded to ImmuCellAI to evaluate immune infiltration, employing the Wilcoxon rank sum test for group comparisons. Additionally, Spearman correlation analysis was conducted to investigate the relationship between hub clock-associated DEGs and various immune cell types.

### Drug target prediction

The DGIdb website (https://www.dgidb.org/) was utilized to explore the relationships between drugs and their corresponding gene targets. The results from this analysis were then used to identify potential therapeutic agents that target hub clock-associated DEGs.

### Establishment of a ceRNA network

MicroRNAs (miRNAs) and long non-coding RNAs (lncRNAs) linked to intersection genes were identified using the miRNet2.0 online database (https://www.mirnet.ca/), facilitating the creation of a comprehensive network of mRNA-miRNA-lncRNA interactions. Additionally, the competing endogenous RNA (ceRNA) network was visualized using Cytoscape software (version 3.8.2).

### Statistical analysis

All statistical analyses were conducted with R software. For comparisons between two groups, normally distributed variables were analyzed using Student’s *t*-tests, while non-normally distributed variables were evaluated using Mann–Whitney *U*-tests. Categorical variables, expressed as percentages, were compared via the Chi-Square test or Fisher's exact test. Spearman correlation analysis was employed to assess relationships. Statistical significance was set at a p-value of less than 0.05.

## Results

### Detection of clock-associated DEGs

**Fig. 1:**
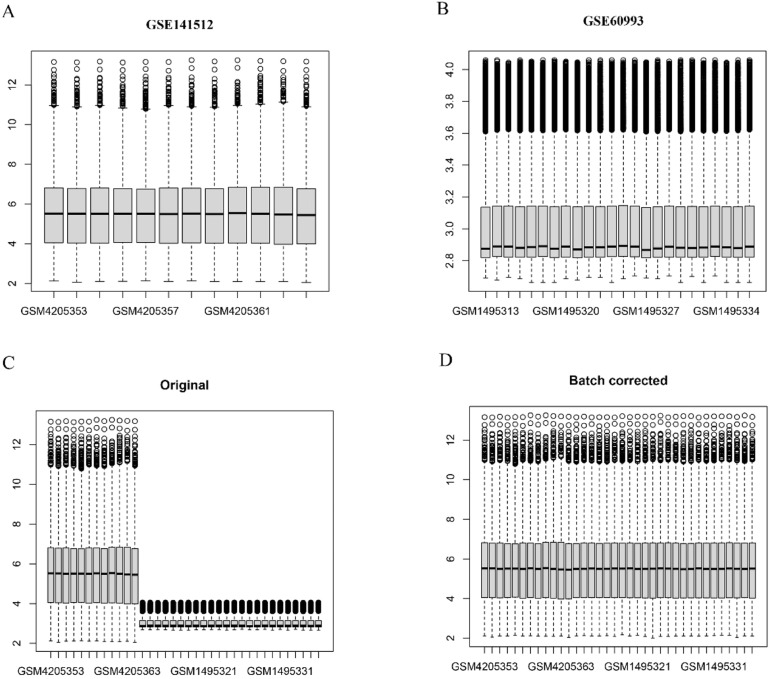
(A‒B) The box plots of gene expression in MI-related transcriptome datasets GSE141512 and GSE60993. (C‒D) Batch correction results of the merged dataset from GSE141512 and GSE60993 before and after correction.

**Fig. 2:**
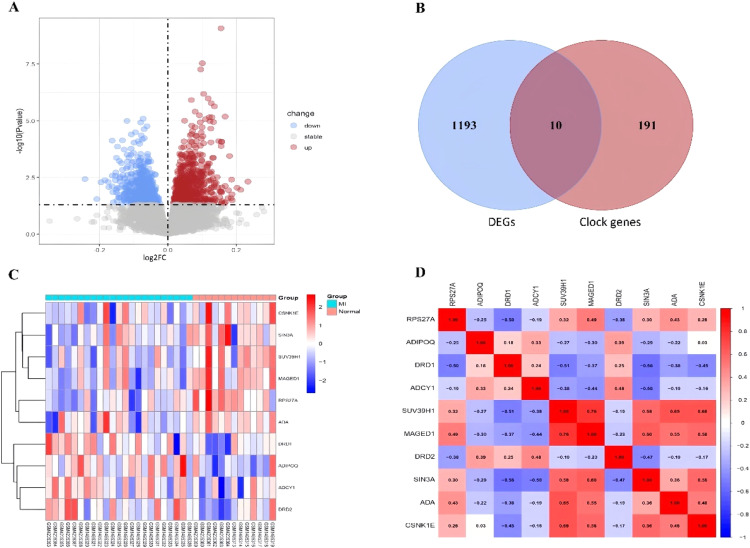
(A) Volcano plot of DEGs in the merged dataset, Upregulated genes are marked in light red; downregulated genes are marked in light blue. (B) The two datasets showed an overlap of 10 DEGs. (C) Heat maps of differentially expressed clock genes in MI and Control. (D) Interrelationships among differentially expressed clock genes.

The authors retrieved transcriptome datasets related to MI, specifically GSE141512 and GSE60993, from the GEO database. These datasets were integrated and underwent batch correction (Fig. 1A‒D). A total of 1203 DEGs were identified in the comparison between MI patients and the control group, with 651 genes showing upregulation and 552 exhibiting downregulation. The DEGs were visualized using a volcano plot (Fig. 2A). Additionally, the authors sourced 201 circadian clock-related genes from the GSEA website, among which 10 were found to overlap with the identified DEGs (Fig. 2B). To analyze the expression patterns of these clock-associated DEGs across the different samples, the authors created a clustering heatmap (Fig. 2C), revealing significant variability. Further correlation analysis was performed to explore relationships among these genes (Fig. 2D), leading to several key observations: RPS27A demonstrated a positive correlation with MAGED1 and ADA, but a negative correlation with DRD1. SUV39H1 also demonstrated positive correlations with MAGED1, SIN3A, ADA, and CSNK1E. Additionally, MAGED1 was positively associated with SIN3A, ADA, and CSNK1E. Conversely, SIN3A showed negative correlations with DRD1, ADCY1, and DRD2, whereas ADA was negatively linked to DRD1. Lastly, CSNK1E was negatively correlated with DRD1.

### Analysis of functional roles of clock-associated DEGs

**Fig. 3:**
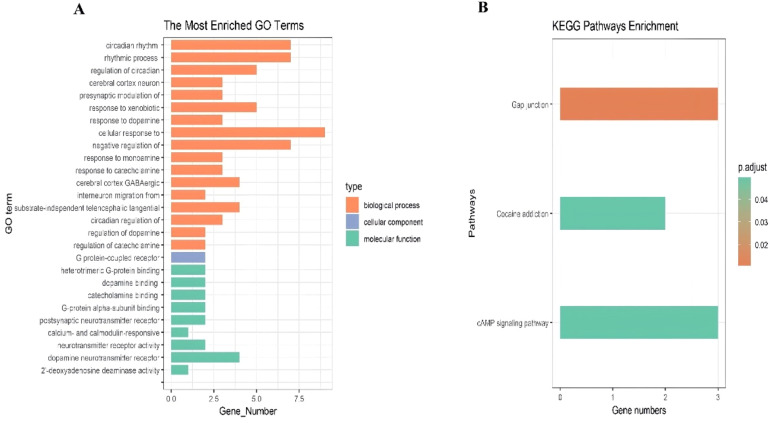
(A‒B) The GO terms and KEGG pathways which is enriched by the differentially expressed clock gene.

The clock-associated DEGs underwent GO and KEGG pathway enrichment analyses. The GO analysis indicated significant enrichment in biological processes, particularly in the cellular response to dopamine and rhythmic processes (Fig. 3A). Similarly, the KEGG pathway analysis demonstrated notable enrichment in the Gap junction and cAMP signaling pathways (Fig. 3B).

### Identification of hub clock-associated DEGs

**Fig. 4:**
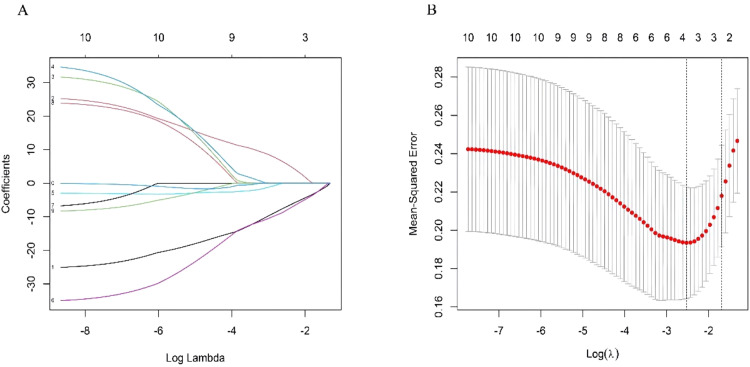
(A‒B) Screening differentially expressed clock genes: 2 genes are screened by Lasso regression method.

**Fig. 5:**
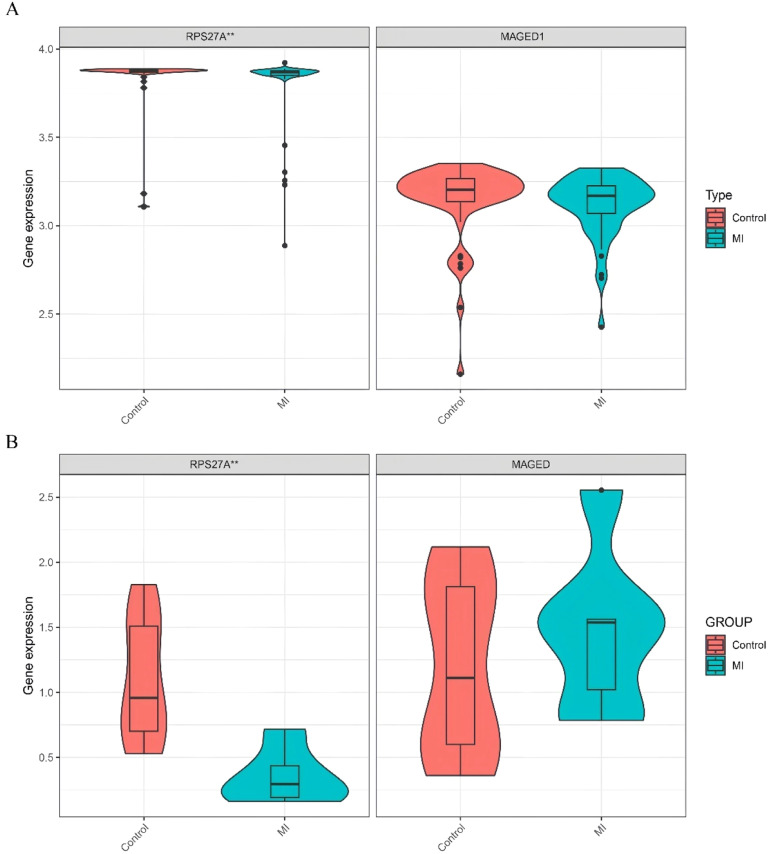
(A) Expression of the marker genes in the validation dataset (GSE66360). (B) Quantitative Reverse Transcription Polymerase Chain Reaction (qRT-PCR) analyses of the expression levels of RPS27A and MAGED1 in peripheral blood samples isolated from controls (*n* = 10) and MI patients (*n* = 6), *p < 0.05; **p < 0.01; ***p < 0.001.

Using the training datasets GSE141512 and GSE60993, the authors performed Lasso regression on the expression matrix of ten clock-associated DEGs. This analysis identified RPS27A and MAGED1 as promising candidates (Fig. 4A‒B). To confirm these findings, the authors analyzed the GSE66360 validation dataset. The results indicated a significant reduction in RPS27A expression levels in the MI group compared to the control group, while MAGED1 expression levels did not display significant differences between MI patients and healthy controls (Fig. 5A).

### Levels of RPS27A and MAGED1 expression in peripheral blood samples

The study comprised 6 MI patients and 10 controls matched for age and gender, with demographic characteristics detailed in Table S2. Analysis of mRNA expression levels indicated that MAGED1 did not show significant differences between MI patients and the control group. Conversely, RPS27A expression was significantly reduced in the MI group compared to the controls (Fig. 5B, Table S3).

### Single-gene GSEA analysis

**Fig. 6:**
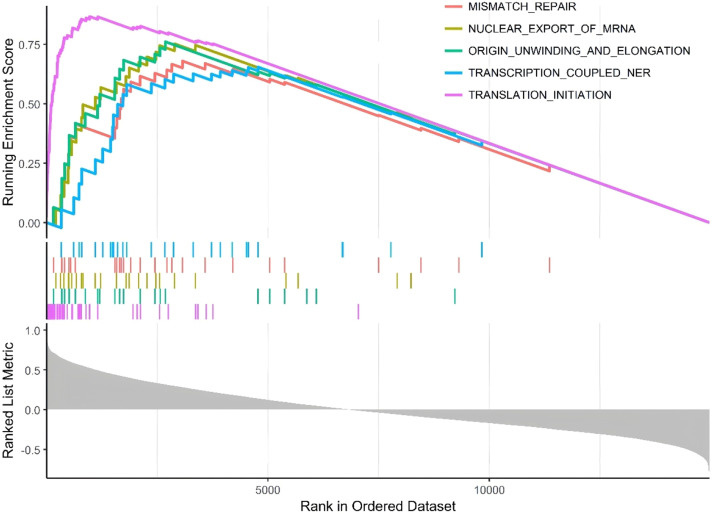
The top five GSEA enrichment analysis of RPS27A.

single-gene GSEA was conducted on the hub clock-associated DEGs to investigate relevant pathways (Fig. 6). The findings revealed that RPS27A was significantly enriched in pathways associated with mismatch repair and the nuclear export of mRNA.

### Investigation of immune cell infiltration

**Fig. 7:**
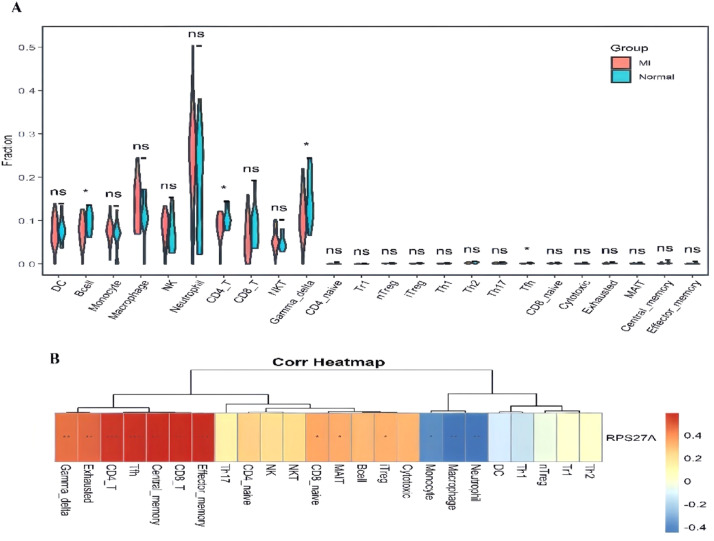
(A) The analysis of immune cell infiltration. (B) Correlation between immune cells and RPS27A, *p < 0.05; **p < 0.01; ***p < 0.001.

The analysis of immune cell infiltration demonstrated significant differences in the levels of B-cells, CD4+ T-cells, gamma delta T-cells, and T-follicular helper (Tfh) cells when comparing the MI group to the control group (Fig. 7A). Furthermore, the relationship between RPS27A expression and various immune cell populations was illustrated (Fig. 7B). RPS27A showed a strong positive correlation with CD4+ T cells, CD8+ T-cells, Tfh cells, gamma delta T-cells, and effector memory cells. In contrast, it exhibited a negative correlation with monocytes, neutrophils, and macrophages.

### Identification of potential therapeutic agents

**Fig. 8:**
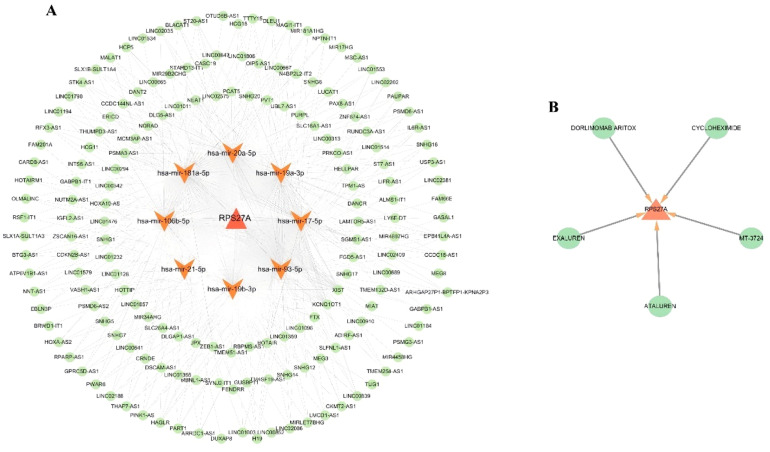
(A) To predict the potential effective therapeutic drugs for RPS27A. (B) ceRNA regulatory network. LncRNA, miRNA and mRNA interactions regulating of RPS27A.

The DGIdb database was used to discover potential therapeutic agents aimed at the hub clock gene RPS27A associated with Myocardial Infarction (MI). (Fig. 8A). This analysis identified five promising therapeutic agents for RPS27A: EXALUREN, ATALUREN, MT-3724, CYCLOHEXIMIDE, and DORLIMOMAB ARITOX.

### Network of ceRNA regulation

A competing endogenous RNA (ceRNA) network was constructed for the clock gene RPS27A, comprising 180 nodes, which included 1 DEG, 10 miRNAs, and 169 lncRNAs, along with 545 edges, illustrating the complex interactions within this network (Fig. 8B). Several lncRNAs were identified as competing for binding with specific miRNAs: 73, 72, 62, 46, 46, 72, 24, and 72 lncRNAs were linked to hsa-miR-106b-5p, hsa-miR-17-5p, hsa-miR-181a-5p, hsa-miR-19a-3p, hsa-miR-19b-3p, hsa-miR-20a-5p, hsa-miR-21-5p, and hsa-miR-93-5p, thereby influencing RPS27A expression. Additionally, LINC02086 was identified as a key regulator of RPS27A expression through its interactions with hsa-miR-106b-5p, hsa-miR-17-5p, hsa-miR-20a-5p, and hsa-miR-93-5p

## Discussion

Recent years have seen a concerning rise in the prevalence and mortality rates linked to coronary heart disease, especially MI. Disturbingly, this trend is increasingly impacting younger individuals, including those in professional roles. Studies have shown that disruptions in circadian rhythms, such as participating in nocturnal activities or working long night shifts, can substantially raise the risk of myocardial infarction.^10^ These findings are particularly significant among younger populations.

Circadian rhythms have gained significant attention as researchers explore their influence on various health aspects. Organisms have developed circadian rhythms synchronized with a 24-hour cycle to adjust to the Earth's environment, managing daily activities and physiological variations.^11^ Disruptions in these rhythms have been linked to a range of diseases, including sleep disorders, cancer, and cardiovascular conditions.^12^ Given this, many researchers speculate about the role of circadian dysfunction in the development of MI or heart attacks. Studies have shown a strong correlation between working night shifts and increased susceptibility to myocardial reperfusion injury and microcirculatory disorders in patients with acute MI.^13^ Additionally, specific circadian patterns have been identified in the onset of symptoms, delays in seeking medical care, and the timeliness of reperfusion treatment in patients with ST-elevation myocardial infarction.^14^ These circadian rhythms are closely tied to the initiation of symptoms, microcirculatory function, and the occurrence of myocardial reperfusion injury in MI.

The significance of circadian clock-related genes in MI has recently attracted considerable attention. These clock genes, which are expressed across various cell types, are regulated by neurons located in the brain's suprachiasmatic nucleus.^15^ Numerous studies have consistently shown a strong association between clock genes and the onset and symptoms of coronary heart disease.^16^ Moreover, Genome-Wide Association Studies (GWAS) have underscored the significance of genetic factors in the development of MI.^17^ Previous studies have primarily concentrated on the roles of well-known circadian clock genes, such as BMAL1, CLOCK, and PER, in the context of MI, often overlooking lesser-known but potentially significant circadian clock genes.^18^ To date, no studies have employed high-throughput transcriptome datasets to explore the circadian clock genes associated with MI pathogenesis. In this study, bioinformatics techniques were utilized to examine the differential expression of clock genes in MI patients compared to healthy controls. Several clock genes were identified as differentially expressed, including ADA, ADCY1, CSNK1E, DRD1, DRD2, SIN3A, RPS27A, SUV39H1, ADIPOQ, and MAGED1. These findings could provide an essential foundation for future animal studies or research involving human samples.

Enrichment analysis of the differentially expressed clock genes indicated significant participation in biological processes, particularly regarding the cellular response to dopamine and rhythmic activities. Dopamine, a neurotransmitter and precursor to noradrenaline, is crucial for cardiovascular and renal functions. It enhances myocardial contractility and cardiac output, facilitates both passive and active vasodilation, and promotes diuresis and natriuresis.^19^ Research has demonstrated that dopamine exhibits circadian-like behaviors in various brain regions, including the retina, olfactory bulb, striatum, midbrain, and hypothalamus, where it both influences and is influenced by clock genes.^20^ This interplay suggests that clock genes may affect the onset of MI by modulating cellular responses to dopamine. Moreover, KEGG enrichment analysis of the ten clock genes underscored their involvement in pathways such as the Gap junction and cAMP signaling pathways. Gap junctions are essential for determining cell survival versus cell death across various cell types, and some aspects of reperfusion injury in the heart have been attributed to gap junction mediation.^21^ Identifying these pathways offers a novel perspective for exploring the role of clock genes in myocardial infarction.

LASSO regression is commonly employed to identify risk factors for diseases and to develop predictive models. While effective in identifying significant variables, this approach does not allow for causal interpretations. Therefore, it is essential to meticulously evaluate the study design and the quality of the data utilized.^22^ In this investigation, LASSO regression was applied to identify key clock genes linked to MI. The reliability of these hub genes, discovered through bioinformatics analysis, was subsequently validated by qRT-PCR, highlighting RPS27A as a potential biomarker.

RPS27A, though not a well-known circadian clock protein, is a ribosomal protein primarily responsible for ribosome biogenesis. Beyond this role, it exhibits biological pleiotropy and participates in mRNA transcription.^23^ While its involvement has been linked to diseases such as cancer, no studies have directly associated it with MI. RPS27A is expressed as a fusion protein with ubiquitin, playing a crucial role in post-translational modifications essential for cellular processes like protein degradation and signaling.^24^ In clear cell renal carcinoma, elevated expression of RPS27A has been linked to the activation of the NF-κB signaling pathway. This activation may enhance inflammation within the tumor microenvironment by regulating the ubiquitin-proteasome system, thereby contributing to tumor progression.^25^ Both NF-κB signaling and ubiquitination have been identified as critical contributors to the pathogenesis of MI.^26^^,^^27^ These findings suggest that RPS27A may influence the development of MI by modulating ubiquitination processes and inflammatory pathways. Research suggests that RPS27A promotes the expression of inflammatory factors in microglial cells by regulating the PSMD12/NF-κB signaling axis, which facilitates immune cell infiltration into brain tissue and exacerbates brain damage in ischemia/reperfusion models.^28^ The potential role of RPS27A in myocardial Ischemia-Reperfusion (I/R) injury through mediating immune cell infiltration remains uncertain, as direct studies on this topic are lacking. The present study on immune cell infiltration demonstrated significant differences in the abundance of B-cells, CD4+ T-cells, gamma delta T-cells, and T-follicular helper (Tfh) cells between patients with MI and healthy controls. Further analysis revealed a strong correlation between RPS27A and various immune cell populations, including CD4+ and CD8+ T-cells. This indicates that RPS27A may play a role in MI by influencing immune responses and circadian rhythms. The observed relationship between RPS27A and immune infiltration in MI suggests that certain clock genes may impact the development of myocardial infarction through their effects on the immune system.

The results of the GSEA analysis revealed the upregulation of several pathways in MI, including mismatch repair and nuclear export of mRNA. Recent studies suggest that DNA mismatch repair plays a significant role in the development and progression of Cardiovascular Disease (CVD). Accumulating DNA damage with age can induce cell death, promoting the formation of unstable plaques, which can lead to adverse cardiovascular events.^29^ Furthermore, mismatch repair and circadian rhythm have been implicated in the functional mechanisms of the TIMELESS gene in relation to carcinogenesis.^30^ This suggests that RPS27A may influence the onset of MI by regulating the mismatch repair pathway. Recognizing these pathways provides crucial insights for further investigation into the functions of genes associated with MI.

This study successfully identified candidate drugs that target the hub gene RPS27A and established a ceRNA network. Epigenetic mechanisms, particularly those involving non-coding RNAs, are vital in the onset and progression of MI, significantly impacting these processes. The present analysis revealed that various lncRNAs are predicted to competitively interact with miR-106b-5p, miR-17-5p, miR-181a-5p, miR-19a-3p, miR-19b-3p, miR-20a-5p, miR-21-5p, and miR-93-5p, thereby regulating the expression of RPS27A. Recent research indicates that miR-106b-5p and miR-19b-3p may serve as early biomarkers for MI.^31^^,^^32^ while miR-17-5p, miR-181a-5p, miR-19a-3p, miR-20a-5p, miR-21-5p, and miR-93-5p have demonstrated significant clinical utility in managing this condition.^33^, ^34^, ^35^, ^36^

Among the potential therapeutic agents identified were EXALUREN, ATALUREN, MT-3724, CYCLOHEXIMIDE, and DORLIMOMAB ARITOX. ATALUREN is a novel, orally administered drug designed to target nonsense mutations. It is approved by the European Medicines Agency for treating Duchenne Muscular Dystrophy in patients aged 5 years and older who can walk.^37^ MT-3724 is an engineered toxin body that targets CD20 and is primarily used for treating non-Hodgkin's lymphoma.^38^ CYCLOHEXIMIDE is an antibiotic produced by the bacterium Streptomyces griseus. It serves multiple roles, including acting as a bacterial metabolite, a protein synthesis inhibitor, a neuroprotective agent, and a ferroptosis inhibitor. It is used as a fungicide and has anticancer properties.^39^ The drugs identified in this study predominantly consist of anti-tumor agents, reflecting a growing trend in utilizing these medications for investigating heart diseases. This trend may indicate shared pathogenesis between cardiac conditions and tumors. However, the effectiveness of the predicted gene-targeted drugs and non-coding RNAs remains uncertain, highlighting the need for further research to clarify the specific pathways involved. As a result, these identified drugs and non-coding RNAs should be prioritized in future studies.

### Limitations

The findings of this study are based on a comprehensive secondary analysis of publicly available data. However, several limitations should be acknowledged. First, the absence of clinical information for certain samples in the GEO database may have introduced biases in the bioinformatics analyses. Additionally, the experimental validation cohort is limited to a single-center Chinese population with a relatively small sample size, which restricts the generalizability of these findings. To enhance external validity, future studies should incorporate multi-center cohorts with diverse ethnic backgrounds, enabling more robust and broadly applicable conclusions. This study establishes only an association between RPS27A and MI, suggesting its potential as a biological marker for MI. However, it does not demonstrate PRS27A as a causal gene in the pathogenesis of MI. This represents a limitation of the study, and further extensive basic research is needed to determine whether PRS27A has a causal role in MI development. Investigating the functional mechanisms of PRS27A will not only provide greater biological insight into these findings but also strengthen their potential for clinical application.

## Conclusion

In summary, the bioinformatics analysis has identified ten potential clock-related genes linked to MI, with RPS27A standing out as a critical regulator of circadian rhythms. These findings underscore the significant role of RPS27A in the pathogenesis of MI, deepening the understanding of the disease and paving the way for promising therapeutic interventions.

## Ethics approval and consent to participate

This study was supported by the Natural Science Foundation of Xinjiang Uygur Autonomous Region, China (Grant No. 2022D01C8). The ethical approval for this research was obtained under approval n° XYDWFYLSH-2022028. Although the project has been completed, all patient data used in this study were collected during the project implementation period under the aforementioned ethical approval number (approval n° XYDWFYLSH-2022028).

## Availability of data and materials

The datasets used and analyzed during the current study are available from NCBI GEO: GSE141512, GSE60993 and GSE66360.

## Authors’ contributions

Conception and design of the research: Rui Xu, Guiqiu Cao; Acquisition of data and Analysis and interpretation of the data: Rui Xu, Changshun Yan; Statistical analysis: Rui Xu, Changshun Yan; Writing of the manuscript: Rui Xu; Critical revision of the manuscript for intellectual content: Rui Xu.

## Fundings

This study was supported by the Natural Science Foundation of Xinjiang Province of China (2022D01C8).

## Conflicts of interest

The authors declare no conflicts of interest.

## Appendix Supplementary materials

Image, application 1
