# Supplementary material for: RPS27A as a potential clock-related diagnostic biomarker for myocardial infarction: Comprehensive bioinformatics analysis and experimental validation
Source: Clinics (Sao Paulo). 2025 May 22;80:100677. doi: 10.1016/j.clinsp.2025.100677 (PMC12148814; doi:10.1016/j.clinsp.2025.100677)
Supplement: Supplementary file 1 [file mmc1.docx]

CLINICS-D-24-01220_Supplementary Materials

**Additional File: Table S1** Primer sequences used for qRT-PCR.

| **Genes** | **Forward** **primer sequence** | **Reverse primer sequence** |
| --- | --- | --- |
| RPS27A | TCGTGGTGGTGCTAAGAAAA | TCTCGACGAAGGCGACTAAT |
| MAGED1 | TGCCTTCTTCGTCAAGCCCC | CCGCACCACAGTCCATTTTCT |
| β-actin | GGACTTCGAGCAAGAGATGG | AGCACTGTGTTGGCGTACAG |

**Additional File: Table S2** Demographic characteristics of MI patients and controls.

| **Variables** | **MI** | **Control** | **p-value** |
| --- | --- | --- | --- |
| Case | 6 | 10 |  |
| Age (years) | 59.33±4.71 | 56.5±14.60 | 0.582 |
| Gender (male) | 3 | 7 | 0.607 |

**Additional File: Table S3** Relative expressions of the plasma RPS27A and MAGED1 in MI patients and controls.

| **Variables** | **MI** | **Control** | **p-value** |
| --- | --- | --- | --- |
| Case | 6 | 10 |  |
| RPS27A | 0.30 (0.17, 0.54) | 0.96 (0.62, 1.55) | 0.002 |
| MAGED1 | 1.53 (0.84, 1.81) | 1.11 (0.55, 1.88) | 0.428 |

Data were presented as median (interquartile range).
